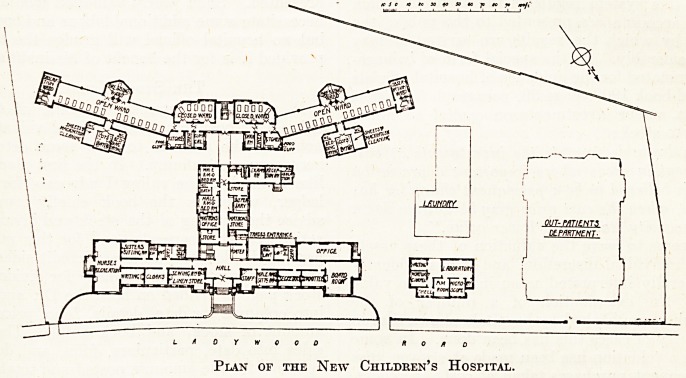# The New Children's Hospital, Birmingham

**Published:** 1913-06-21

**Authors:** 


### HOSPITAL ARCHITECTURE AND CONSTRUCTION.

### The New Children's Hospital, Birmingham.

This hospital has been planned on somewhat novel lines. It comprises at present three distinct blocks: (*a*) the administration block, (*b*) the ward block, and (*c*) the pathological block. A laundry and an out-patient department are to be added at some future time.

The administration block is five storeys in height. On the ground floor are, to the right, the secretarial offices, board and committee rooms. To the left, sewing room, nurses' cloak room, writing room, and recreation room, and sisters' sitting room, with lavatory accommodation. In the back wing on one side are quarters for male resident medical officer; on the other, stores and dispensary. A small projecting building at the south-west end contains a reception and examination room for patients, isolation ward, and bath room. On the first and second floors are the quarters for the female medical officers, matron, nurses, and servants, and on the third floor are the kitchen offices.

It is in the planning of the ward block that the novel element comes in. In the centre is the main staircase and lift, with a small ward for five cots on each side towards the south-west, while on the north-east are two ward kitchens, a dark room, linen store, and clinical room. On either side of this centre block long wards project with their axes bent, so as to make an obtuse angle with the axis of the centre part. These long wards have only one row of beds, and the outer wall to the south is so constructed that it can practically be cleared away altogether and the ward becomes an open-air one. In the centre of each wing is a small semi-octagonal ward, capable of being warmed to a high temperature, and designed for the purpose of changing dressings. At the further end of each wing is an observation ward, with an escape staircase adjoining. The sanitary offices are placed in projecting towers on the north side.

The wings of this block are three storeys in height, and the total accommodation for patients is 138. The centre part is carried up one storey higher, and contains the operation department.

The pathological block contains a waiting room, mortuary chapel, *post-mortem* room, and two laboratories.

The hospital was erected from the designs and under the supervision of Mr. F. W. Martin, of Birmingham.

Plan of the New Children's Hospital.

## Figures and Tables

**Figure f1:**